# Analysis of Vibrations During Trochoidal Milling of AZ31 Magnesium Alloy Using Different Tool Holders

**DOI:** 10.3390/ma19214564

**Published:** 2026-10-29

**Authors:** Jarosław Korpysa, Ireneusz Zagórski, Andrzej Weremczuk

**Affiliations:** 1 Department of Production Engineering, Lublin University of Technology, Nadbystrzycka 38D, 20-618 Lublin, Poland; i.zagorski@pollub.pl; 2 Department of Applied Mechanics, Lublin University of Technology, Nadbystrzycka 38D, 20-618 Lublin, Poland; a.weremczuk@pollub.pl

**Keywords:** AZ31 magnesium alloy, trochoidal milling, vibration, P-P, RMS, CMSE, FFT, recurrence analysis

## Abstract

This paper presents the analysis of vibration measurements performed on AZ31B alloy for different tool holder types under trochoidal tool paths. The principal aim of the study was to determine the influence of selected technological parameters (cutting speed *v*_c_ and feed per tooth *f*_z_), together with tool holder type (ER, shrink-fit and hydraulic), on selected measures of machining stability. The analysis encompasses the Peak-to-Peak (P-P) value, the Root Mean Square (RMS) value, Composite Multiscale Entropy (CMSE), the Fast Fourier Transform (FFT) and recurrence plots (RP) analysis. The results demonstrate that vibration characteristics depend on both machining parameters and tool holder type. Increasing feed per tooth generally increases vibration amplitude, whereas CMSE provides complementary information on signal irregularity and the relative contribution of periodic and irregular components. Frequency-domain and recurrence analyses reveal differences in the dynamic response of the investigated tool holder systems. The hydraulic holder generally exhibits lower vibration amplitudes and less pronounced harmonic components, consistent with its favorable damping characteristics. Considering the measured vibration amplitudes and complementary signal characteristics, cutting speeds of 400 and 800 m/min are recommended under the investigated conditions. The results also demonstrate the value of combining amplitude-based and nonlinear signal analysis for a more comprehensive assessment of machining dynamics.

## 1. Introduction

Modern machine tools are essentially provided with two types of spindle tips and dedicated tool holders. The most widely used standards are ISO (7/24 taper) and HSK (Hollow Shank Kegel, 1/10 taper). The advantage of 7/24 taper tool shanks is their symmetrical design and ease of manufacture, which contributes to their low cost. They are well-suited for less demanding machining operations. Unfortunately, their shortcoming is the possibility of axial tool displacement due to the expansion of the spindle taper at high rotational speeds, resulting from the deformation of the tool holder under the influence of the centrifugal force. The same phenomenon also reduces the contact between the tapered surfaces. At high rotational speeds, there is also a risk that the tool length preset with a setting device may change. Another drawback relates to the protruding drive keys which can become loose due to the action of centrifugal forces and thus pose a significant hazard. For these reasons, this clamping method should not be used at speeds exceeding approximately 15,000 rev/min. A solution designed to mitigate the negative effects of high rotational speeds is the HSK with a 1/10 taper. Its design offers numerous advantages over conventional 7/24 taper shanks. This taper is more precise thanks to its front contact surface; it is stiffer, facilitates the transmission of high bending moments and torques, and does not require the use of additional clamping devices. Other advantages of the HSK system include: the ability to operate at high spindle speeds even exceeding 40,000 rev/min; high static and dynamic rigidity of the tool-to-spindle connection; reduced weight and length of the tool and tool holder; high vibration stability of the machine tool–holder–workpiece–tool (MHWT) system; reduced chatter occurrence; high accuracy and repeatability of the connection; reliable torque transmission; simple operation; and quick retooling [1]. Therefore, it is important and essential to select the appropriate machine tool right at the beginning of the production process planning, taking into account, for example, the high spindle speed ranges achievable with HSK spindle tips, the use of high-speed electric spindles, and HSK precision tool holders.

Another interesting factor that can affect the outcome and effect of machining is the type of tool holder itself. The most popular and widely used tool holders in the machine tool industry include ER-type tool holders (tool holders with so-called spring collets, used at lower rotational speeds and cutting speeds), shrink-fit tool holders, and hydraulic tool holders. The use of a heat-shrink tool holder also makes it possible to balance the tool holder together with the cutting tool to ensure a high-quality machined surface, minimize machining vibrations, and reduce the centrifugal force, resulting in axial runout of the tool. Among hydraulic tool holders, one can distinguish Shunk’s design solutions such as Tribos and Tendo (SCHUNK SE & Co. KG, Lauffen n. Neckarem, Germany). These are advanced tool holding systems that ensure both high holding precision and effective damping of machining vibrations. These features contribute to longer tool life (especially when machining difficult-to-machine materials), as well as ensure high-quality machined surfaces. The Tendo hydraulic tool holder provides, among other things, the highest tool holding force while simultaneously offering high vibration damping. However, users report its slightly lower resistance to tool pull-out during so-called heavy rough machining compared to thermal tool holders. This holder is specifically designed for mechanical engineering applications requiring high accuracy in precision machining. Tool changing in the TENDO system is quick and does not require expensive peripheral equipment (as is the case, for example, with shrink-fit holders). The tool shank is clamped and released manually using a single hex key. As standard, these holders are precision-balanced to class G2.5 at 25,000 rpm, making them ideal for high-speed machining applications. Tools with smaller diameters can be mounted using dedicated reduction sleeves. Special grooves inside the clamping chamber displace residual oil, grease, or coolant, ensuring dry contact and reliable high-torque transmission. In contrast, the Tribos hydraulic tool holder features a slim profile, high vibration damping, and very low axial and radial runout of the tool holder (below 0.003 mm at an overhang of 2.5 × D). The system facilitates quick and easy replacement of cutting tools using special clamping devices. Under the prevailing polygonal clamping principle, the mounting bore is polygonal in shape when at rest. When subjected to external pressure within a special fixture, the bore becomes perfectly circular, allowing the tool shank to be inserted. Upon release of the pressure, the material returns to its original shape, locking the tool securely and concentrically in place. The internal structure of the holder features a so-called honeycomb design. This unique internal structural characteristic absorbs vibrations generated during the machining process. The only drawback is the need to use so-called peripheral devices for tool clamping; however, the clamping process itself takes place at ambient temperature (whereas shrink-fit holders require heating and cooling). Like TENDO tool holders, TRIBOS tool holders feature a high level of dynamic balance (grade G2.5) and a radial runout of less than 0.003 mm at an overhang of 2.5 × D [2].

Based on their type (from the perspective of the vibration source), vibrations can be classified as natural, forced, and self-excited. Each of these vibration types can be described using simple mechanical models, based on the equation of motion for a vibrating system with one degree of freedom and by comparing the system’s vibration frequency and amplitude. From the perspective of machining, the most interesting is self-excited vibration (chatter) that occurs in the absence of an external exciting force acting on the system. The occurrence of these vibrations is associated with a loss of stability in the machine tool–holder–workpiece–tool (MHWT) system. Among the many causes of instability, one can identify the following two that are most common: internal coupling within the MDE (mass–dissipative–elastic) system and the machine tool–holder–workpiece–tool (MHWT) system; and coupling via so-called trace machining or trace regeneration (vibration reproduction) [3]. Stability diagrams, also known as stability lobe diagrams (SLDs), play a significant role in chatter prevention methods. These diagrams clearly visualize the boundaries between the stable machining region, below the stability lobe curve, and the unstable region—above the curve. One can also distinguish the region of absolute stability, the depth of cut below which the process is stable regardless of the rotational speed, and the regions of relative stability, located between the curves. Stability diagrams make it possible to determine the limit values of the depth of cut in relation to the rotational speed, with the depth of cut considered to be the most important technological parameter affecting the milling process stability. Given that the curves are generated while the machine tool is at a standstill—and thus do not account for all factors, including spindle rigidity, temperature variations, and factors related to the workpiece (e.g., weight, material defects)—it is important to bear in mind the limited accuracy of this method [4,5].

A study into key parameters affecting the stability in milling [6] made it possible to describe the effect of parameters such as radial and axial depth of cut, spindle speed (rotational speed), feed rate, and tool geometry. The study used the FLN method (Minis’ Fourier analytics) and the convolution force model to predict vibration stability. As the tool diameter, stiffness, and damping ratio were increased (or as the cutting force and radial depth of cut were decreased), the SLD curve tended to move upward. In contrast, as the natural frequency was increased, the SLD curve would tend to move to the right. With an increase in the number of teeth, the SLD curve would tend to move downward. Among the methods for improving the stability, or vibration stability, of machine tools and cutting tools, one can distinguish the following: design changes (adding damping elements or increasing their stiffness), the use of vibration absorbers, and the modification of the track regeneration effect (disrupting phase coincidence between adjacent vibration waves). In addition, various techniques are employed to increase dynamic stiffness and vibration stability (for long tool overhangs), including: the use of anisotropic shanks, the use of materials with a high Young’s modulus and high damping, the use of so-called passive dynamic absorbers, and the use of active vibration control (active vibration dampers) [3,7].

A considerable number of studies investigated machining vibrations during milling components, including thin-walled, made primarily of aluminum alloys, such as EN AW-2014-T6 [8], EN AW-2017A [9], EN-AW 2024 [10], EN AW-6061-T6 [11,12], EN AW-6063 [13], as well as the popular alloy EN AW-7075-T7451 [14,15,16,17,18]. For example, the trochoidal milling of EN AW-2014-T6 [5] was conducted with parameters of the so-called stable SLD region (*n* = 5026 rev/min; *a_p_* = 0.6 mm). The measured vibration displacement signal in the *x*-axis direction decreased over the course of the machining process. In contrast, when using parameters from the unstable region (*n* = 4586 rev/min; *a_p_* = 1.4 mm), greater vibration fluctuations were observed, which, unfortunately, did not become stable. For the 2017A alloy, however, the numerical value of the vibration was (at *n* ≈ 6400 rev/min, *a_p_* = 1–3 mm) approx. 2 m/s^2^ at the lowest depth of cut and approx. 15 m/s^2^ at the highest depth of cut [9].

An attempt to determine the optimum machining parameters was also made using the EN AW-6063 alloy and various machining conditions (with n up to 3000 rev/min, f up to 0.06 mm/rev, *a_p_* and a_e_ ranging from 1.5 to 3.5 mm) [13]. An increase in *f* resulted in a decrease in the amplitude of vibration acceleration (to a value of approx. 0.8 m/s^2^), whereas for a combination of *n*, *a_p_* and *a_e_*, the use of their medium range values proved to be the most favorable. It was also demonstrated that the helix angle had the greatest influence on process stability; increasing the angle to 45° resulted in a significant reduction in vibration levels, from approximately 5 m/s^2^ to 1 m/s^2^. A subsequent study on the EN AW-7075 alloy [14] showed the dominance of vibrations along the *x*-axis in down milling, whereas vibrations along the *y*-axis prevailed in up milling. In both cases, the greatest machining stability and best surface quality were achieved at the highest value of *n* = 6000 rev/min and *f_z_* = 0.01 mm/tooth. The low peak values of vibration acceleration along the *y*-axis and the narrow range of variation suggest higher stiffness in the machining direction along this axis. Research on a thin-walled component made of the 7075-T6 alloy was also conducted in [15]. The vibration signal was analyzed, and the so-called nonlinear stability criterion was determined based on the phase plane method, Poincaré method, and spectral analysis. Changes in the depth of cut and spindle speed, as well as their effect on the maximum Lyapunov exponent, were analyzed.

Vibration analyses are not limited to selected types of light alloys; they can also include materials that are difficult to machine, such as titanium alloys [19,20], nickel alloys [21], and steel [22,23,24]. For example, the application of protective coatings on tool holders in steel machining [23] significantly reduced vibration displacement, with the epoxy coating being the most effective and yielding the best results when combined with AlCrN- and TiAlN-coated tools. Within the considered range of machining parameters, the lowest vibration displacement amplitude of 0.014 mm was obtained for a combination of an FBE-coated tool holder and an AlCrN-coated end mill, at *n* = 3500 rev/min and *f* = 0.2 mm/rev. A simulation of steel machining (including steels with alloying elements such as Ni, Cr, and Mo) [24] confirmed the significant influence of tool holder type on the stability of the milling process conducted with rotational speeds up to 10,000 rev/min, *f_z_* = 0.04 mm/tooth, and *a_p_* = 0.2 mm. The use of a hydraulic tool holder achieves up to 3-fold-lower vibration displacement values in the *x*-axis (approx. 45 μm) and *y*-axis (approx. 11 μm), compared to a shrink-fit tool holder and an ER collet chuck tool holder (90–130 μm in the *x*-axis and 25–40 μm in the *y*-axis, respectively).

The magnitude of vibration, especially chatter, can also depend on many other factors. In addition to the type of material being machined and the type of tool holder, it is important to use modern types of tool paths or machining strategy [25]. Modern tool paths certainly include the trochoidal path, which offers a number of advantages over the straight path [26,27], or the epicycloidal path [28]. Apparently, the trochoidal path outperforms in many aspects conventional milling, as well as epicycloidal milling, exhibiting lower values of vibration and cutting force components [17]. At the same time, epicycloidal tool paths significantly reduce the machining time (by up to 15%) due to shorter curve paths. Trochoidal milling utilizes circular motions combined with forward feed. Epicycloidal tool paths are defined by complex mathematical curves consisting of a plane curve traced by a fixed point on a circle, rolling without slipping around the rim of another, stationary circle.

One of the more recent studies in this field proposed solutions involving the modification of an ER-type tool holder to incorporate sensors for measuring and analyzing cutting force and vibrations. While this solution facilitates contactless data transmission, it requires modifications to the machine tool’s spindle housing. The application possibilities were tested using the S355JR grade steel as an example. Among other things, the occurrence of so-called chatter marks and the values of the force and torque signals were analyzed [29]. Such a solution may prove effective for machining materials at low rotational and cutting speeds. Another study investigated the influence of sensorized tool holders (conventional ER, without sensors; sensorized tool holder of SPIKE type with a multi-lock chuck; sensorized tool holder of RCD type) on the cutting force *F_x_*, maximum width of the flank wear land (*VB_max_*) and *Sa* surface roughness parameter. In addition to that, a modal analysis was performed. The tool holders were mounted on the tip of an HSK A63 spindle. The sensorized tool holders can be applied without negative effects on machining processes subject to compliance with the machine tool–holder–workpiece–tool (MHWT) system’s modal characteristics. Concluding, the configuration of the SPIKE type tool holder yielded the highest dynamic compliance, while the dynamic compliance of other configurations was about half lower [30].

The review of the literature devoted to vibration and tool holders reveals a research gap regarding the problem of magnesium alloy machining. There is a steady increase in the use of various milling methods, both in domestic and global production, which makes milling the most promising machining method. Advances in machine tools, cutting tools, machining strategies and techniques expand the potential applications of milling. Significant advances in CNC machines also contribute to both increased productivity and machining efficiency; nevertheless, the occurrence of vibration, particularly chatter, remains a significant limitation. The presence of vibration can reduce both the quality of the machined surface and the stability of the machining process. For these reasons, further research must be conducted in this area. The review of the literature on vibration occurrence in the milling of magnesium alloys stresses the need to expand research in this area. Studies conducted to date have focused on the use of straight tool paths and various tool holder configurations [26,27]. Basically, only one study has investigated the problem of trochoidal milling tool path with different tool holder types [31]. There exist single works related to, e.g., the micro-milling of AZ31B [32], but this machining process can hardly be compared with the cutting process conducted using tools with greater diameters. Although the above-mentioned study used a micro-milling machine with a high-speed spindle (up to 80,000 rev/min), the tool diameter was only 1 mm, with two flutes and a cutting edge radius of 1 µm. It was found that the vibration frequency of vibration-assisted micro-milling could affect the machining mechanism, including reduced specific cutting force, tool wear and burr formation. A valuable supplement to these various works is a recent study investigating the machinability of the AZ31B alloy, using different tool holder types and trochoidal milling tool paths. The novelty of this work lies in the application of a trochoidal tool path during the machining of the AZ31B alloy. The authors’ research focuses on the machinability of magnesium alloys, specifically regarding the assessment of milling process stability. The processing of results, such as composite multiscale entropy or recurrence plots, for the machinability analysis of magnesium alloys should be considered innovative. Future analyses could involve visualizing result dispersion using box plots and employing metrics that facilitate a quantitative description of recurrence plots. Apart from the standard vibration analysis (maximum values, peak-to-peak, amplitude, root mean square value), one can conduct an advanced analysis encompassing multiscale entropy, FFT analysis and recurrence plot analysis. Such studies are virtually nonexistent. A vast number of works focus on other structural materials from the group of light metals, such as aluminum alloys and titanium alloys. Furthermore, such works most often address only a single parameter of vibration characteristics, namely vibration amplitude. This approach seems highly insufficient for a comprehensive, complex and detailed description of vibrations that occur in various machining processes. The literature review, therefore, indicates a strong need to extend and broaden the knowledge in this area.

## 2. Materials and Methods

**Figure 1:**
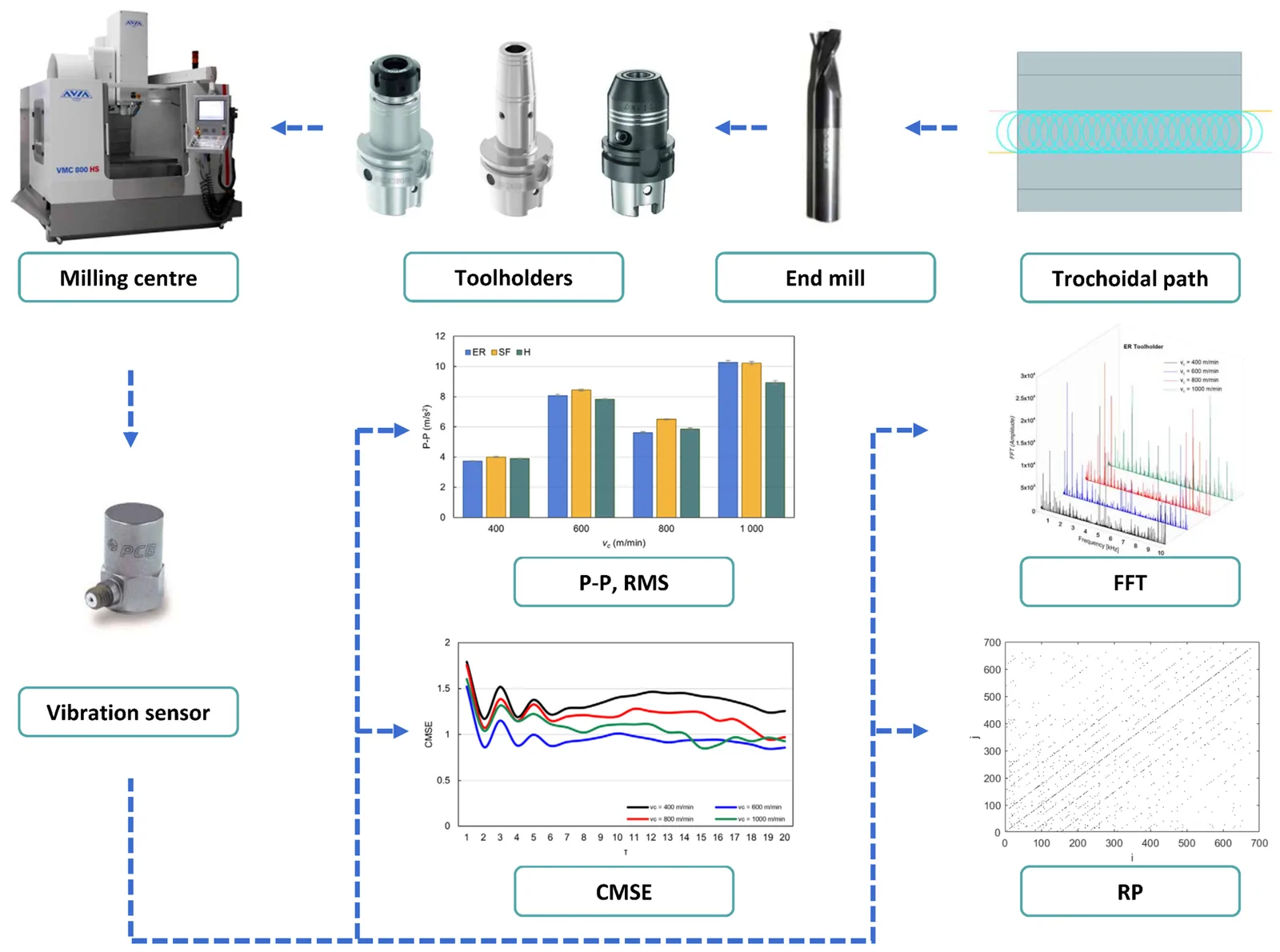
Study design.

Experiments were conducted on AZ31 magnesium alloy, with all milling trials performed on an AVIA VMC 800 HS (Warsaw, Poland) machining center. Three HSK-A63 tool holder types (ER collet, shrink-fit, hydraulic) were selected to evaluate the effect of clamping method on the process. A two-flute, TiAlN-coated carbide end mill (16 mm diameter) was used throughout, following a trochoidal milling strategy generated in Siemens NX, with the trochoidal step set at 15%, corresponding to 2.4 mm (Figure 1).

Two parameters were varied across the test matrix: cutting speed (*v_c_* = 400–1000 m/min) and feed per tooth (*f_z_* = 0.05–0.30 mm/tooth). The axial depth of cut (*a_p_* = 6 mm) and slot width (*b* = 25 mm) were kept constant in all trials. This parameter range was chosen based on three considerations: the kinematic limits of the machine’s spindle, findings reported in earlier studies on magnesium alloy machining, and the stability boundaries derived from Stability Lobe Diagram analysis.

Vibration was monitored using a PCB 352B10 accelerometer (Beijing, China), mounted rigidly at the center of the workpiece’s front face. The vibration acceleration signal was recorded by a sensor oriented along the machine’s *Y*-axis, which coincided with the feed direction. Data were recorded at a frequency of 10 kHz. The duration of the analyzed signal was not constant. When low feed rates were used, the machining time increased, which in turn resulted in a longer signal recording duration. Conversely, as the feed rate was increased, this duration became shorter. This was due to the use of a fixed measurement path length. Vibration signals were evaluated using two quantitative measures: Peak-to-Peak (P-P) value and Root Mean Square (RMS) value.

Signal complexity was further assessed using the Composite Multiscale Entropy (CMSE) algorithm, which is a measure of the signal’s disorder. Its theoretical background and computational procedure are described in detail in [20,33]. The analysis was performed for a scale factor *τ* = 50, whilst the analysis was limited to *τ* = 1–20 due to the greatest variations within this range. The measurement signal was analyzed using standard FFT analysis. The results are presented in the frequency domain, covering the range up to 10 kHz. Subsequently, the measurement signals were analyzed using recurrence algorithms. A standard approach was adopted, basing each plot on 700 points while maintaining constant embedding parameters (*m* = 5, *τ* = 6) and a threshold value *ε* set at 0.75 of the standard deviation. All calculations were performed in Matlab R2019b using previously prepared algorithms [26,33].

## 3. Results

### 3.1. Peak-to-Peak (P-P), Root Mean Square (RMS)

**Figure 2:**
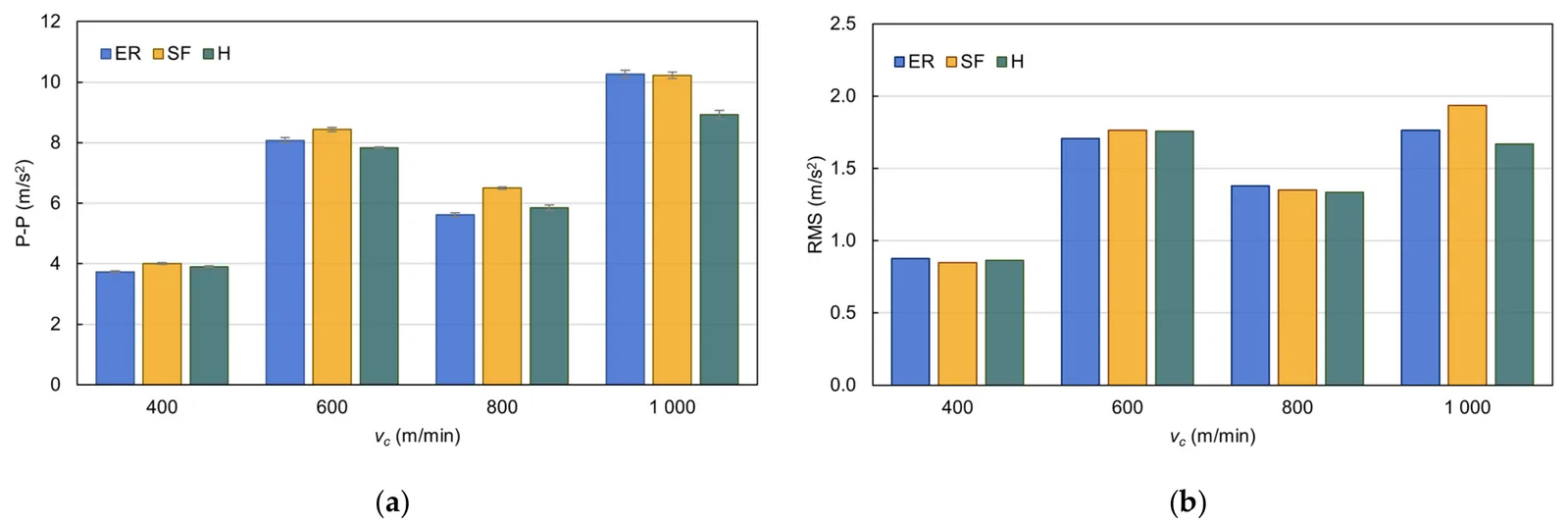
The effect of cutting speed on: (**a**) P-P, (**b**) RMS.

Among the most significant findings of this analysis is the relationship between vibration amplitude and cutting speed (Figure 2), which is clearly non-monotonic for all three tool holder types. In the range of *v_c_* = 400–600 m/min, the P-P amplitude increases sharply by 116.5% for the ER holder (from 3.73 to 8.08 m/s^2^), by 110.4% for the shrink-fit holder (from 4.01 to 8.44 m/s^2^), and by 100.7% for the hydraulic holder (from 3.91 to 7.84 m/s^2^). The RMS value follows essentially the same pattern, increasing nearly twofold irrespective of holder type. A further increase in cutting speed to 800 m/min, reverses this trend: vibration amplitude decreases consistently across all holders, by 23.0–30.5% relative to the maximum recorded at *v_c_* = 600 m/min. At *v_c_* = 1000 m/min, the amplitude increases sharply once again, by 52.7–83.0% relative to the value at *v_c_* = 800 m/min, reaching the highest values recorded across the entire series (P-P of 8.9–10.3 m/s^2^, RMS of 1.67–1.94 m/s^2^). Such non-monotonic behavior indicates that the vibration response depends on cutting speed and may reflect frequency-dependent dynamic amplification within the tool–holder–spindle–workpiece system.

At lower speeds, the differences between holder types are relatively small and not fully conclusive. At *v_c_* = 400 m/min, the spread in P-P values remains below 7.5%, consistent with the fact that, at low excitation energy, the stiffness and damping of the clamping system contribute relatively small to the overall dynamic response. A clear and systematic advantage of the hydraulic holder emerges only at the highest cutting speed tested, where its P-P amplitude (8.92 m/s^2^) is lower by 13.1% than that of the ER holder (10.27 m/s^2^) and by 12.8% than that of the shrink-fit holder (10.23 m/s^2^). Similarly, the RMS value for the hydraulic holder (1.67 m/s^2^) is lower by 5.4% than that of the ER holder and by 13.8% than that of the shrink-fit holder. This indicates that the beneficial effect of the hydraulic holder’s damping capacity is most pronounced under the most severe dynamic loading of the system, that is, precisely when the ability to dissipate vibration energy is of greatest practical importance.

**Figure 3:**
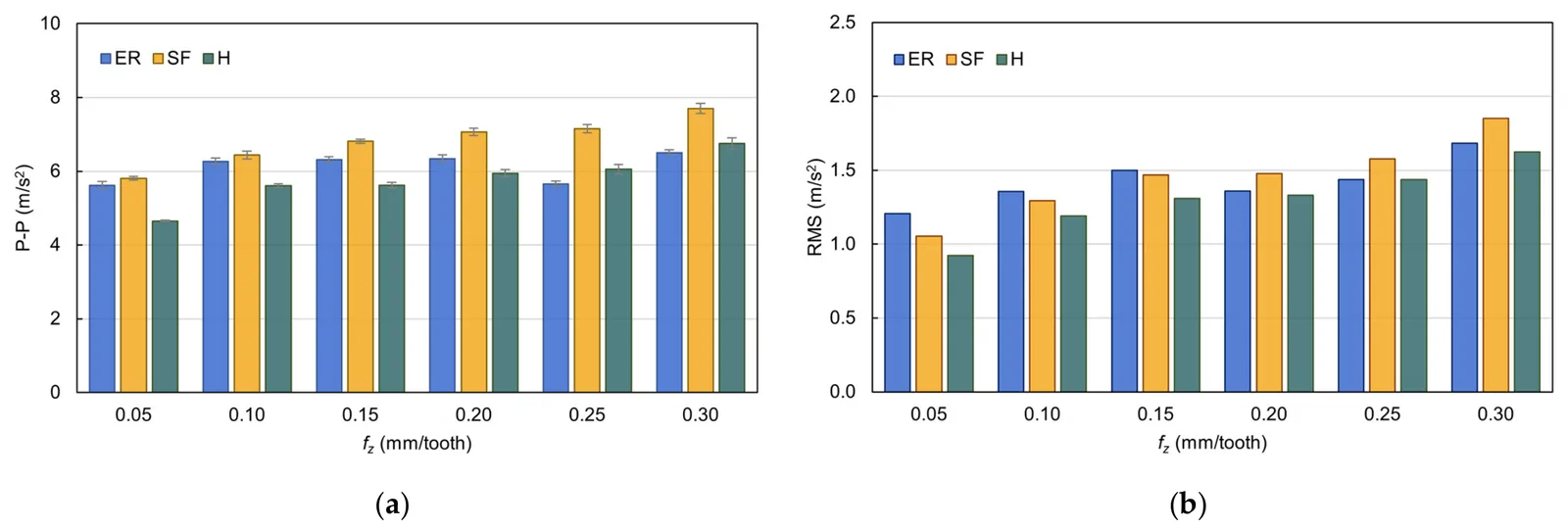
The effect of feed per tooth on: (**a**) P-P, (**b**) RMS.

The dependence of vibration amplitude on feed per tooth (Figure 3) differs markedly from that observed for cutting speed, showing a character close to monotonically increasing for all three tool holder types. Within the range *f_z_* = 0.05–0.30 mm/tooth, the P-P value increases by 15.9% for the ER holder, by 32.6% for the shrink-fit holder, and by 45.1% for the hydraulic holder, while the RMS value increases by 39.6%, 75.6%, and 75.6%, respectively. It is worth noting that, although the ER holder exhibits the highest absolute vibration amplitude at low feed values, its relative sensitivity to an increase in feed is the smallest among the holders studied. The remaining holders, despite lower values in the lower feed range, show a considerably stronger increase in amplitude.

The increase in vibration amplitude with feed results directly from the mechanics of the cutting process: an increase in feed increases the uncut chip thickness, and consequently the magnitude of the excitation force acting on the tool–holder system. The differing relative sensitivity of the individual holders to this mechanism can be attributed to the differing contribution of a baseline vibration component that is not directly related to the cutting force but is instead generated by the clamping system itself. For the ER holder, this contribution is, as also shown by the CMSE analysis, relatively large and only weakly dependent on cutting parameters, which flattens the relative increase in total amplitude with increasing feed. For the shrink-fit and hydraulic holders, in which this component is considerably smaller, the recorded vibration amplitude more directly reflects the cutting force, hence its stronger increase with feed.

The effect of holder type is also considerably clearer and more consistent in the feed series than in the cutting-speed series. The hydraulic holder generates the lowest P-P value across the entire feed range studied, lower than the shrink-fit holder by 12.3–19.9%, and then the ER holder by 6.4–17.1% (with a slight reversal of this relationship at the highest feeds *f_z_* = 0.25–0.30 mm/tooth). RMS values for the hydraulic holder are consistently lower than for the shrink-fit holder by 8.0–12.4% across the entire range, and then for the ER holder by 0–23.3% (this difference decreasing as feed increases, nearly vanishing at *f_z_* ≥ 0.20 mm/tooth). In contrast, the shrink-fit holder generates the highest vibration amplitude at the majority of the measurement points studied.

The significant effect cutting speed, feed per tooth, and tool holder type was also confirmed by an ANOVA statistical analysis—Table 1. For all variables, values of *p* > 0.05 were obtained, confirming statistical differences between the measured vibration values. There is also an interaction between the both cutting parameters and the type of tool holder, indicating that the parameters have different effects depending on the holder used.

### 3.2. Composite Multiscale Entropy (CMSE)

Composite multiscale entropy (CMSE) provides a means of assessing the degree of irregularity, or complexity of a time signal. In the diagnostics of machining-process vibration, a low entropy value indicates a strongly regular signal dominated by a small number of periodic components, whereas a high entropy value indicates a signal with a richer, more stochastic, broadband frequency structure, typical of processes with a significant contribution from random contact and frictional phenomena, or from chaotic instability.

**Figure 4:**
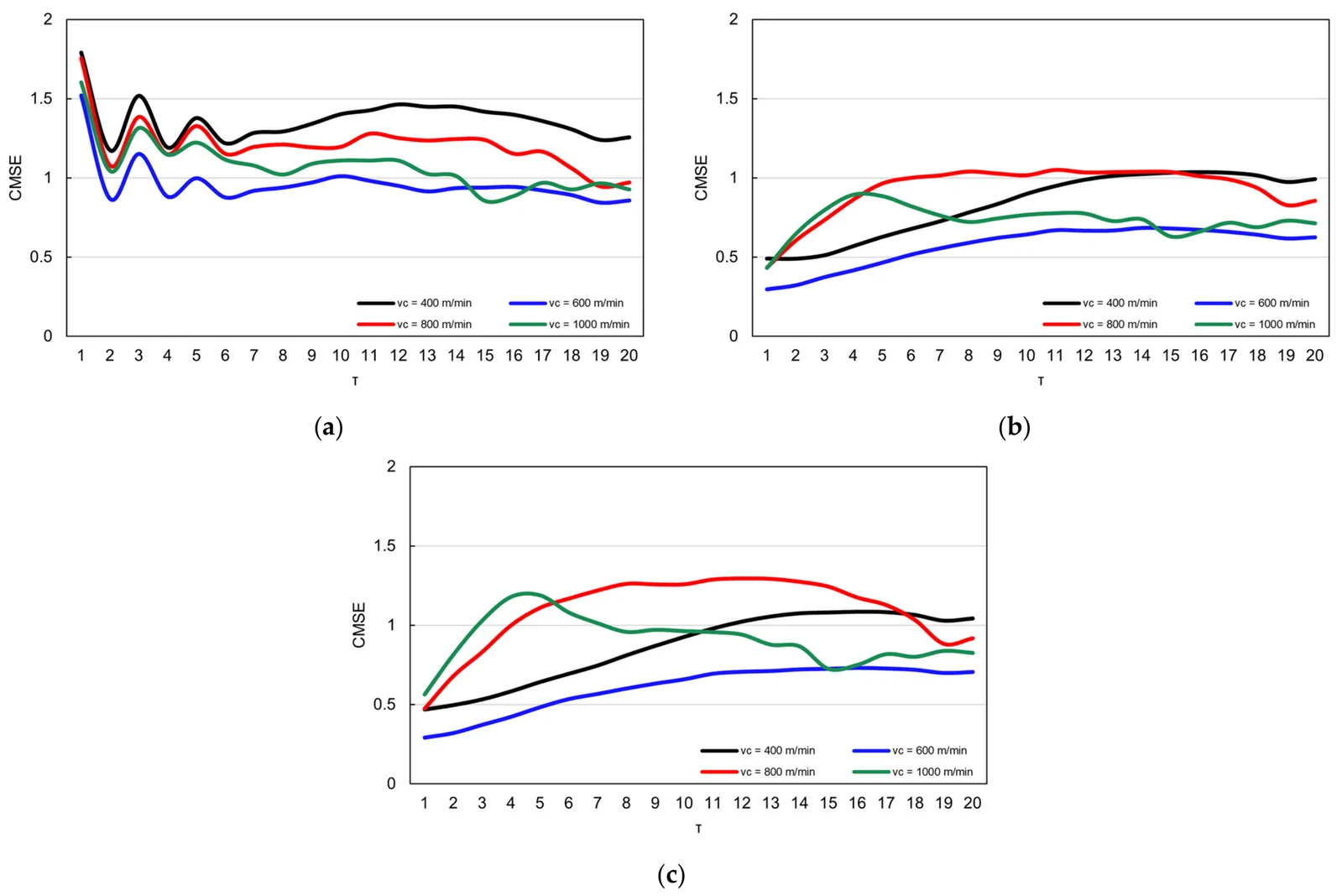
The effect of cutting speed on CMSE during use: (**a**) ER, (**b**) shrink-fit, (**c**) hydraulic holders.

The shape of the CMSE profile as a function of the scale factor (Figure 4) differs qualitatively between the ER holder and the remaining two holders. For the ER holder, regardless of cutting speed, the entropy value is always highest at the smallest scale factor (1.52–1.79) and decreases as *τ* increases, notwithstanding some initial oscillation in the lower range of the scale factor. In contrast, for the shrink-fit and hydraulic holders a different, characteristic hump-shaped course is observed, typical of signals with a multiscale structure. The entropy at *τ* = 1 is relatively low (0.29–0.57), rises with increasing scale, reaches a local maximum typically in the range *τ* = 10–16, and decreases again at the largest scales analyzed. This qualitative difference indicates that, for the ER holder, the dominant contribution to overall signal complexity comes from a fine-scale component that is smoothed out already at low averaging. In contrast, for the other holders significant information about the signal structure emerges only at intermediate time scales, most likely associated with the periodicity resulting from the cyclic nature of tooth engagement with the material. Regardless of holder type, the lowest signal-complexity values are consistently recorded at *v_c_* = 600 m/min, coinciding with a local maximum in P-P and RMS amplitudes relative to the adjacent cutting speeds. This combination indicates a more regular, periodic-dominated vibration response and suggests possible frequency-dependent dynamic amplification. The result illustrates the complementary diagnostic value of CMSE relative to conventional amplitude-based measures.

In addition to the differences related to cutting speed, a persistent, several-fold difference in entropy level between the ER holder and the other holders is also observed across all the conditions studied. At *τ* = 1, the CMSE values for the ER holder (1.52–1.79) are 3–4 times higher than for the shrink-fit (0.30–0.49) and hydraulic (0.29–0.57) holders. This phenomenon can be related to the number and nature of the contact surfaces in the tool-clamping system. The ER collet holder is characterized by a complex, multi-element clamping mechanism in which, under dynamic loading, small, mutually independent micro-displacements may occur, constituting additional components of the recorded signal. The shrink-fit holder is mechanically considerably simpler, which is consistent with its exhibiting the lowest entropy level among the holders studied. The hydraulic holder exhibits an entropy slightly, but consistently, higher than that of the shrink-fit holder, despite showing lower vibration amplitude. A plausible explanation involves the coupled dynamic response of the elastic clamping sleeve and the oil-filled chamber [34]. The compliance of the sleeve and viscous energy dissipation within the hydraulic system may influence the dynamic stiffness and damping of the assembly [35]. This may attenuate dominant periodic components, increasing the relative contribution of low-amplitude, less regular fluctuations to the measured signal and consequently raising CMSE without increasing the overall vibration amplitude. However, the present experiments did not directly measure oil-pressure fluctuations or identify nonlinear fluid–structure interactions. Therefore, the higher CMSE should be interpreted as a possible consequence of the coupled dynamic response of the hydraulic clamping system rather than as direct evidence of nonlinear behavior of the oil layer itself.

**Figure 5:**
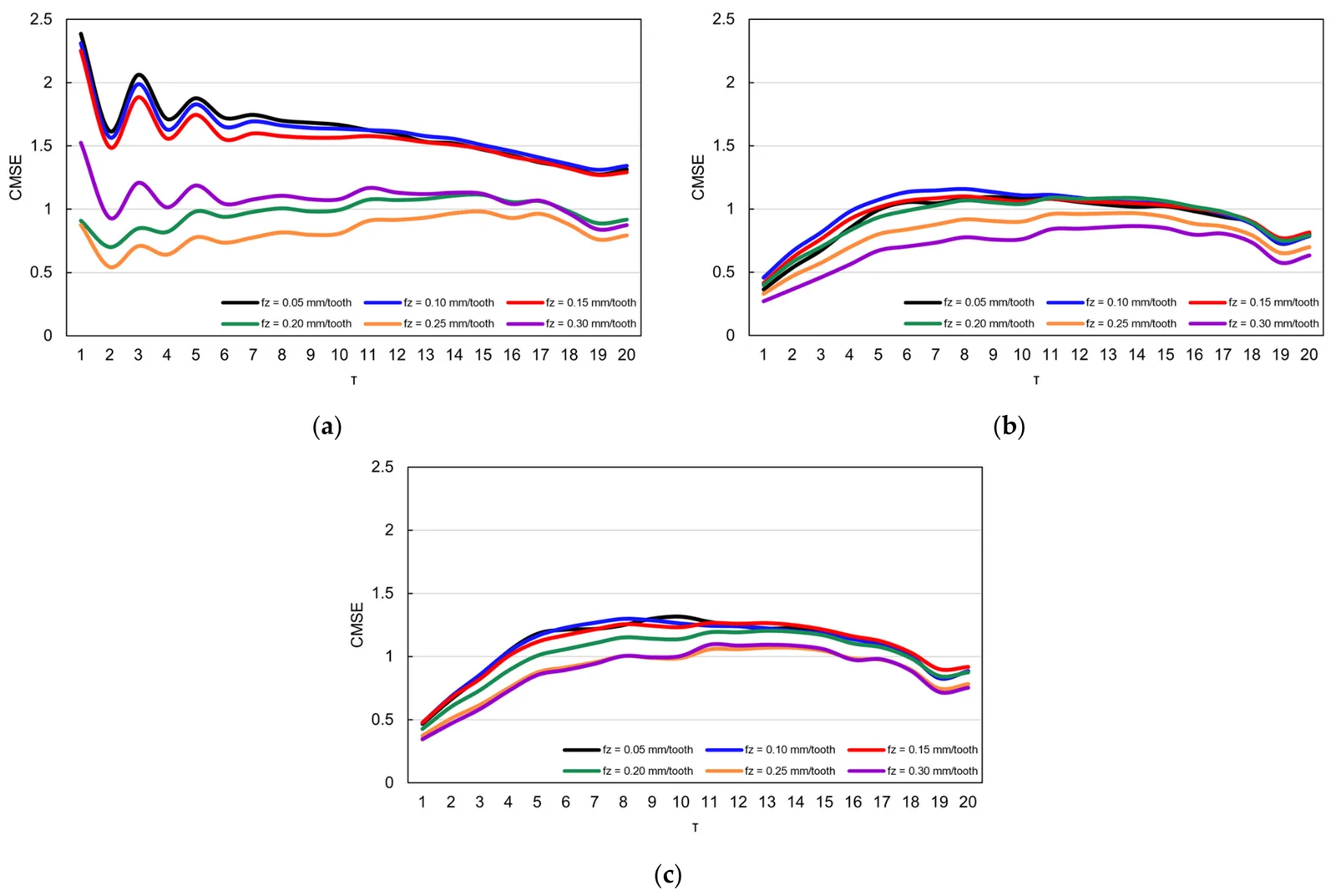
The effect of feed per tooth on CMSE during use: (**a**) ER, (**b**) shrink-fit, (**c**) hydraulic holders.

Similarly to the amplitude measures, the complexity of the vibration signal as a function of feed per tooth (Figure 5), after an initial slight increase or plateau, shows a systematic decrease with further increasing feed. This indicates that the cutting process becomes increasingly dominated by regular, periodic force excitation associated with the increasing thickness of the cut layer, with the relative contribution of stochastic components to the total signal energy decreasing accordingly. This effect is particularly evident for the ER holder. Within the range *f_z_* = 0.05–0.15 mm/tooth, CMSE values remain at a relatively high level (above 1.5), whereas over the upper feed range the entropy value decreases markedly. At low feeds, the amplitude of the force component is small, so the relative contribution of noise generated by the tool–holder system to the total signal is comparatively large, which translates into high entropy at small scales. As feed increases, the amplitude of the periodic force excitation increases and begins to dominate over the relatively constant level of interface noise, reducing its contribution to the signal and thereby decreasing entropy.

The remaining holders exhibit only a modest local maximum around *f_z_* = 0.10–0.15 mm/tooth, followed by a gradual decrease in entropy with increasing feed (by approx. 41% for the shrink-fit holder and 28% for the hydraulic holder). The comparatively low level of baseline vibration noise generated by the clamping system itself means that, even at the smallest feeds studied, the contribution of the cutting-force-related component to the total signal is already relatively large. Its further increase with feed therefore produces only a mild, rather than an abrupt, decrease in entropy.

### 3.3. Fast Fourier Transform (FFT)

Workpiece acceleration signals recorded during the experiments were analyzed in the frequency domain using the Fast Fourier Transform (FFT). This approach enabled the identification of frequency components within the measured acceleration signals. All calculations were performed using pre-developed algorithms in the MATLAB R2019b environment. This facilitated the detection of self-excited vibrations (e.g., chatter), the occurrence of which complicates and significantly hinders the milling process. The analysis focused on workpiece acceleration signals in the feed direction, recorded under varying milling parameters (*v_c_*, *f_z_*) and using three different workholding fixtures. Characteristic peaks visible in the FFT plots indicate specific frequencies of the analyzed acceleration signals and their harmonics. The number of harmonics and their amplitudes can serve to assess the signal’s complexity and, consequently, the complexity of its dynamics. A higher number of harmonics increases the likelihood of their superposition and amplification. The characteristic peaks in the FFT plots arise primarily from the action of individual cutting edges and their interaction with other vibration sources. The superposition of these vibrations can lead to the occurrence of detrimental self-excited vibrations during the milling process.

**Figure 6:**
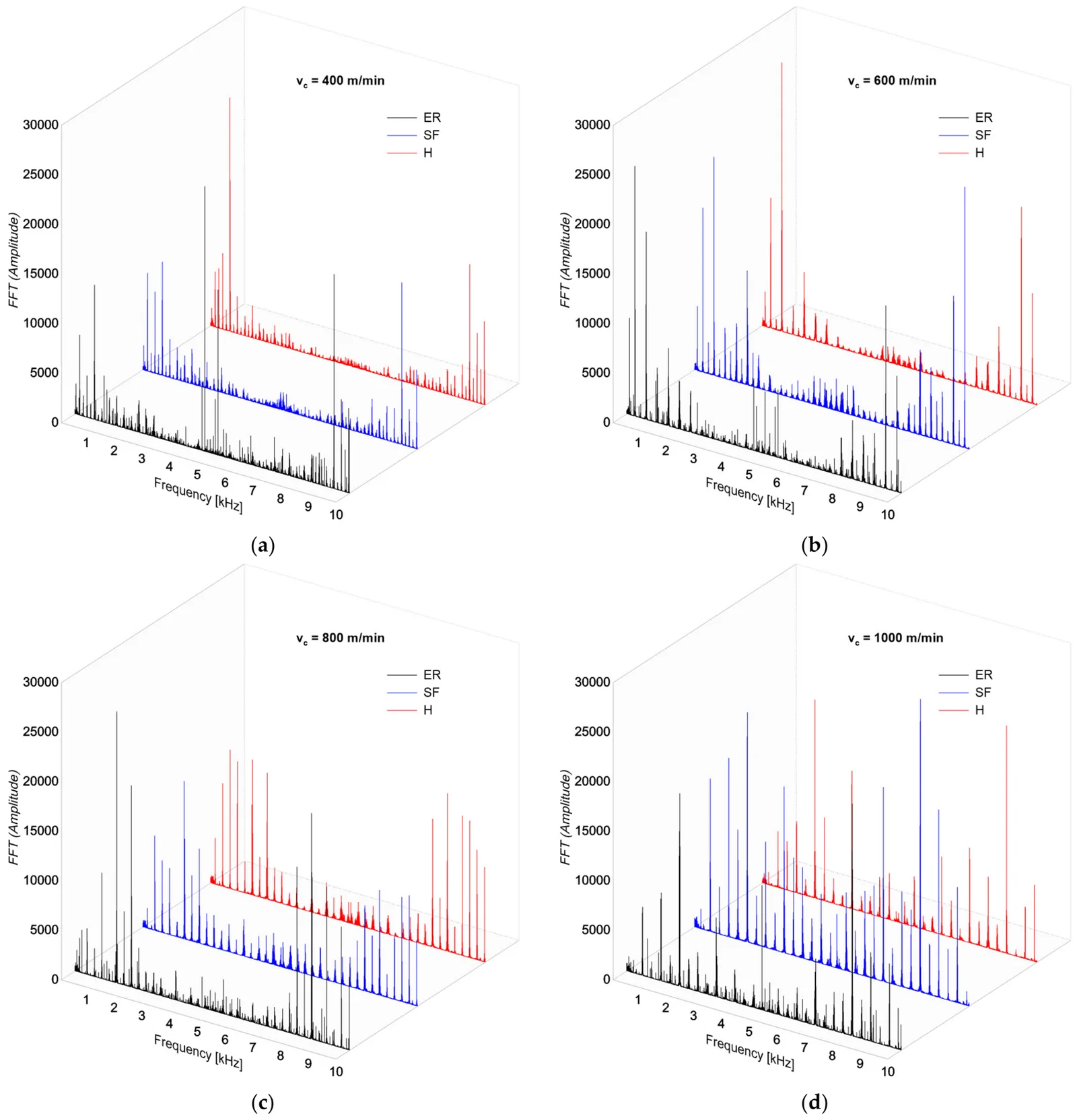
FFT for various tool holders at different milling speeds: (**a**) *v_c_* = 400 m/min, (**b**) *v_c_* = 600 m/min, (**c**) *v_c_* = 800 m/min, (**d**) *v_c_* = 1000 m/min.

Figure 6 presents the FFT analysis results for four cutting speeds. Each plot compares the three tool holders under investigation. At the lowest recorded cutting speed (*v_c_* = 400 m/min), the ER holder exhibited the highest number of harmonics. Two pronounced spectral peaks were observed in the mid-frequency range of 5–6 kHz. Their amplitudes varied with tool holder type and cutting speed, indicating differences in the frequency-domain response of the machining system. In the same frequency range, the other two tool holders showed only a small number of harmonics with very low amplitudes. As the cutting speed increased (*v_c_* = 600 m/min and *v_c_* = 800 m/min), the previously described harmonics in the mid-frequency range were suppressed for the ER holder. An increase in the number of high-magnitude harmonics was also observed at the lower and upper ends of the analyzed frequency range. At a cutting speed of *v_c_* = 1000 m/min, a large number of high-amplitude harmonics appeared for the shrink-fit tool holder. No significant changes in signal structure were observed for the other tool holders compared to the previously tested cutting speeds. A comparison of all results shown in Figure 6 reveals that the most favorable signal profiles were obtained with the hydraulic tool holder. This is likely due to the hydraulic tool clamping mechanism; the presence of oil provides partial damping of machining vibrations, which is particularly evident in the mid-frequency range. This is undoubtedly a beneficial phenomenon for the stability of the milling process.

**Figure 7:**
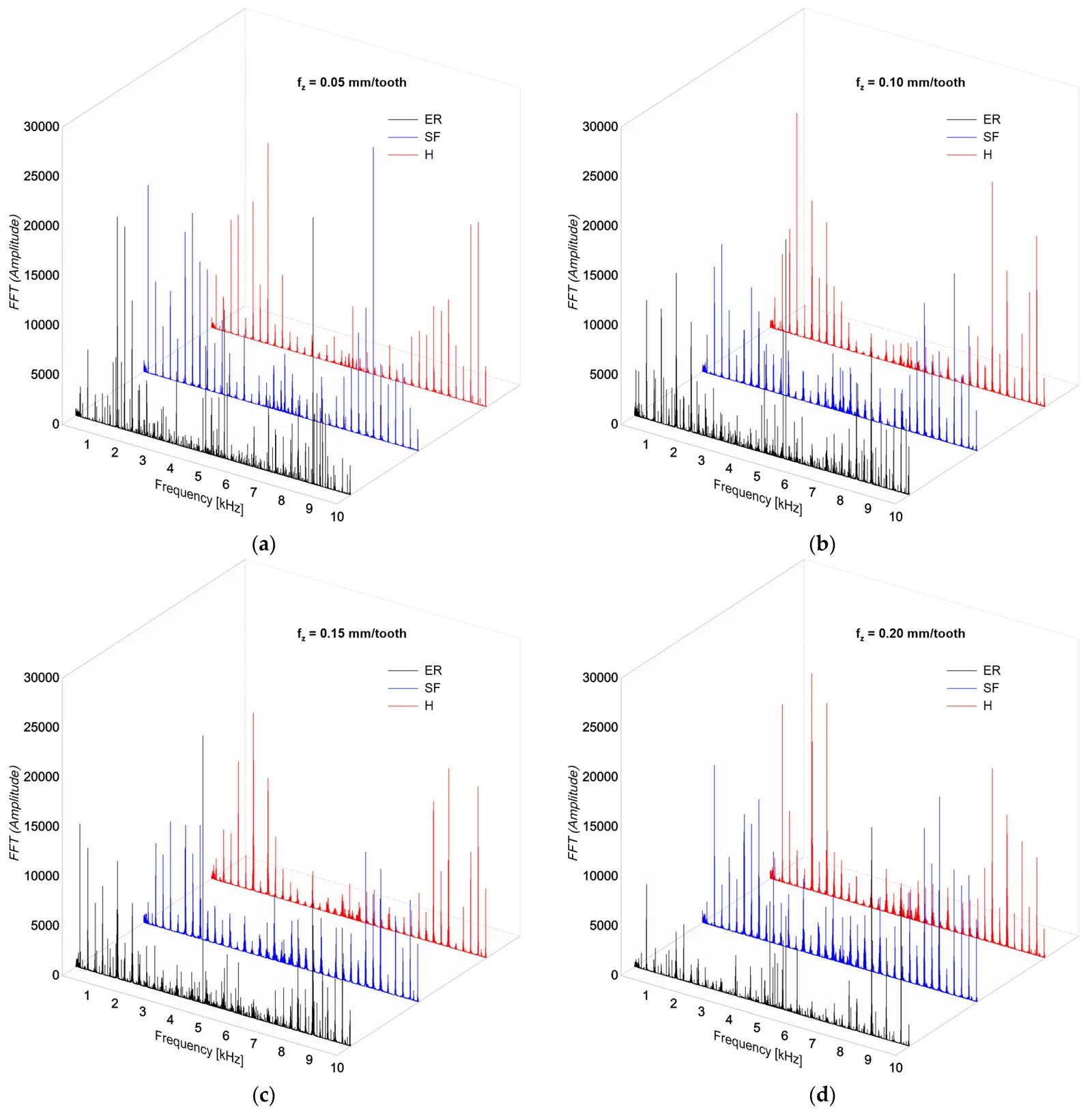

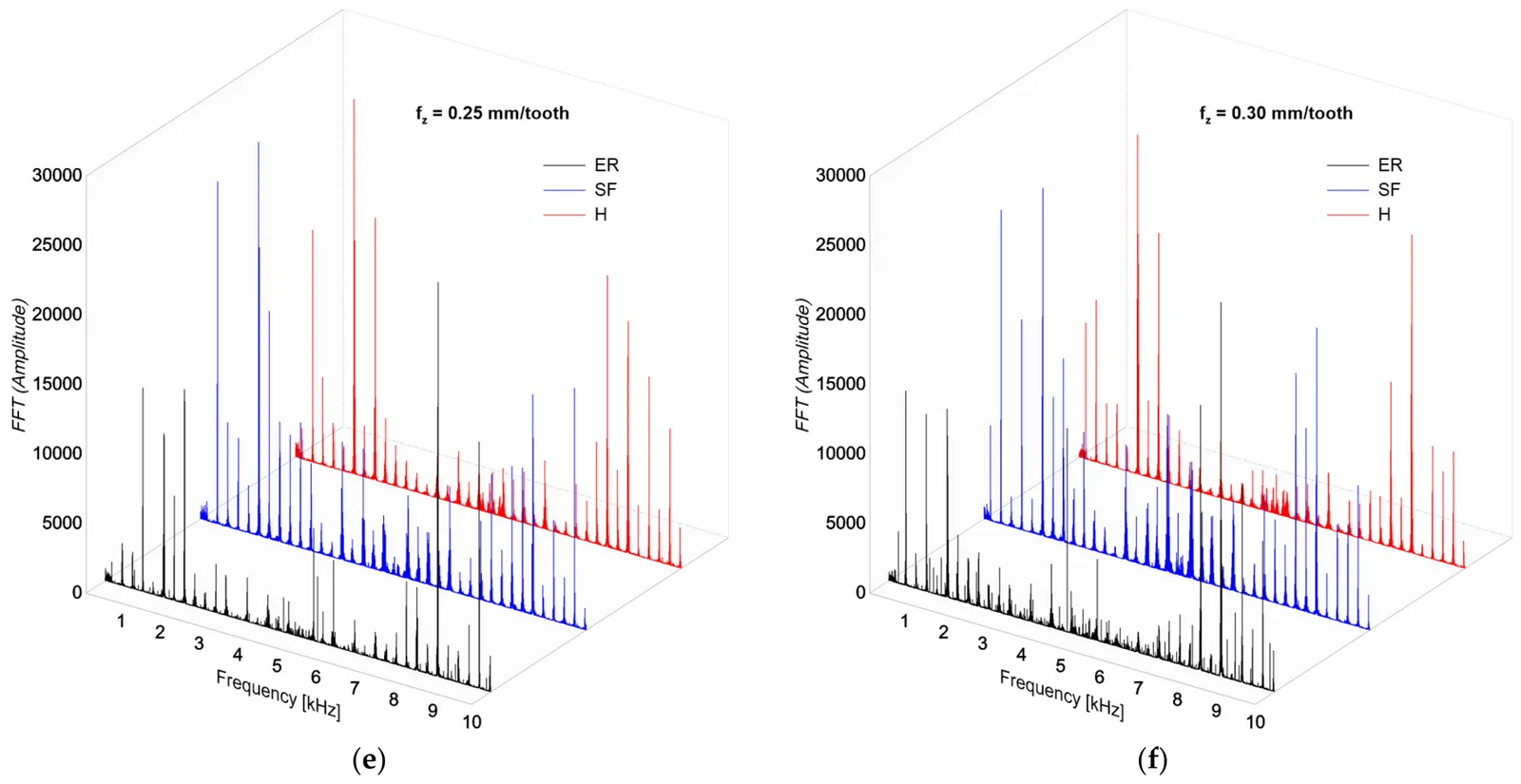
FFT for various tool holders at different feed per tooth: (**a**) *f_z_* = 0.05 mm/tooth, (**b**) *f_z_* = 0.10 mm/tooth, (**c**) *f_z_* = 0.15 mm/tooth, (**d**) *f_z_* = 0.20 mm/tooth, (**e**) *f_z_* = 0.25 mm/tooth, (**f**) *f_z_* = 0.30 mm/tooth.

In the next stage, the effect of the change feed per tooth was verified; the results are presented in Figure 7. A decrease in amplitude values was observed for all tool holders as the feed per tooth increased. This phenomenon persists up to a feed per tooth value of *f_z_* = 0.15 mm/tooth. The gradually increasing thickness of the cut layer stabilizes the machining process to some extent. A greater number of harmonics are generated, but with significantly lower amplitudes. Above a feed per tooth of *f_z_* = 0.2 mm/tooth, the process behavior changes. There is an increase in the number of harmonics, accompanied by an increase in amplitude. This behavior could lead to the onset of dangerous self-excited vibrations beyond the maximum feed value tested (above *f_z_* = 0.3 mm/tooth). A comparison of the results obtained for the three analyzed tool holders reveals that the shrink-fit tool holder is the most susceptible to vibration development. A significantly higher number of harmonics was observed across all traces compared to the other two holders. These harmonics also reach much higher amplitudes than those recorded for the other two holders. As in the previous analysis (regarding the effect of cutting speed), the significant advantage of using a hydraulic tool holder is clearly evident here. Particularly in the mid-frequency range, all plots show fewer harmonics with significantly lower amplitudes. This phenomenon has a beneficial effect on the milling process.

### 3.4. Recurrence Plots (RP) Analysis

Conventional analysis of acceleration signals does not adequately capture the complexity of the milling process. Consequently, a nonlinear recurrence analysis method was proposed; this approach has yielded highly promising results in extensive studies of the dynamics of various measurement signals. A standard approach to analyzing this type of measurement signal was employed in the study. Each recurrence plot was generated using 700 data points, with consistent embedding parameters applied: *m* = 5 and *τ* = 6. The threshold value *ε* was set at 0.75 of the standard deviation. In the study presented here, the plots are arranged so that the upper panel displays the normalized signal corresponding to the recurrence plot (shown in the lower panel).

**Figure 8:**
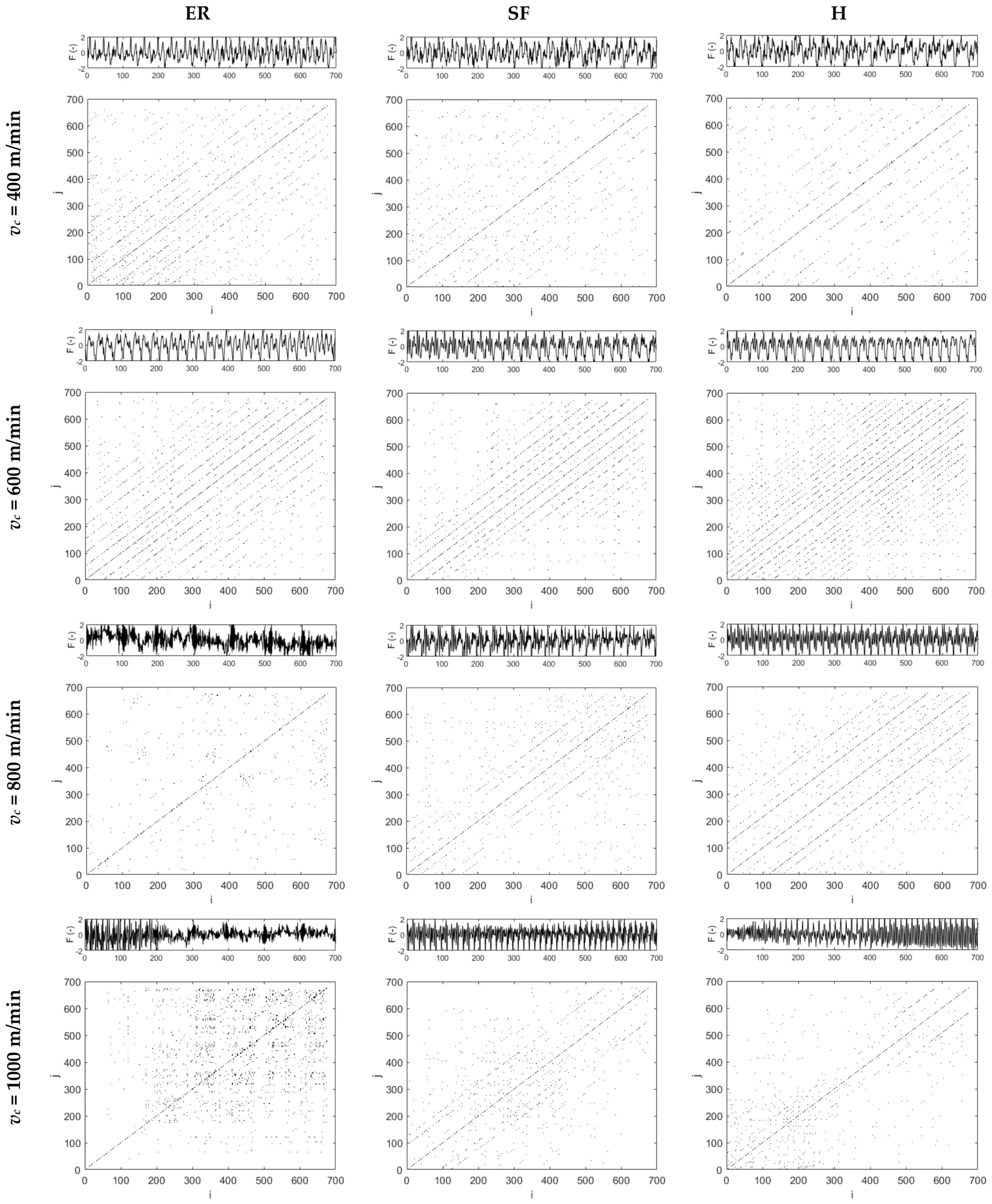
Recurrence plots for various tool holders at different cutting speeds.

Figure 8 present the results of recurrence analysis regarding changes in cutting speed. The columns display results for each of the machining fixtures used. Periodic vibrations were observed in all charts, characterized by very long diagonal lines running parallel to the main diagonal. A direct relationship is also evident between the cutting speed and the spacing of the diagonal lines; increasing the cutting speed results in a shorter force signal period and reduced spacing between these lines. At a cutting speed of *v_c_* = 400 m/min, the diagonal lines appear parallel but exhibit additional fluctuations, as confirmed by the time-domain waveforms. As the cutting speed increases, the lines become more continuous (*v_c_* = 600 m/min), indicating improved stability of the machining process. Further increases in cutting speed reveal a gradual change in the nature of the milling process. In addition to periodic vibrations, extra fluctuations appear, signaling a transition toward a less stable and predictable machining regime. These changes are particularly noticeable at a cutting speed of *v_c_* = 1000 m/min with the ER tool holder.

**Figure 9:**
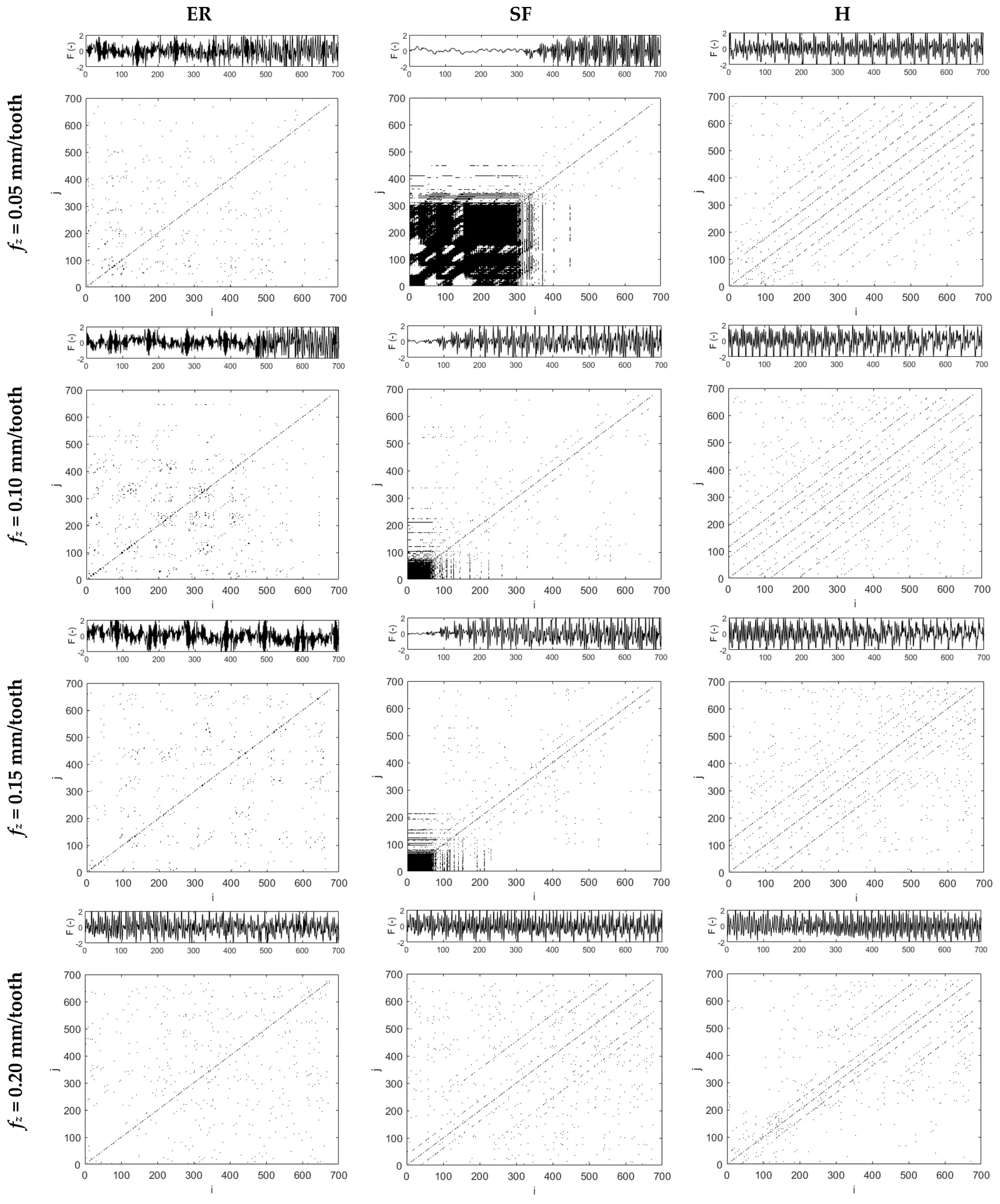

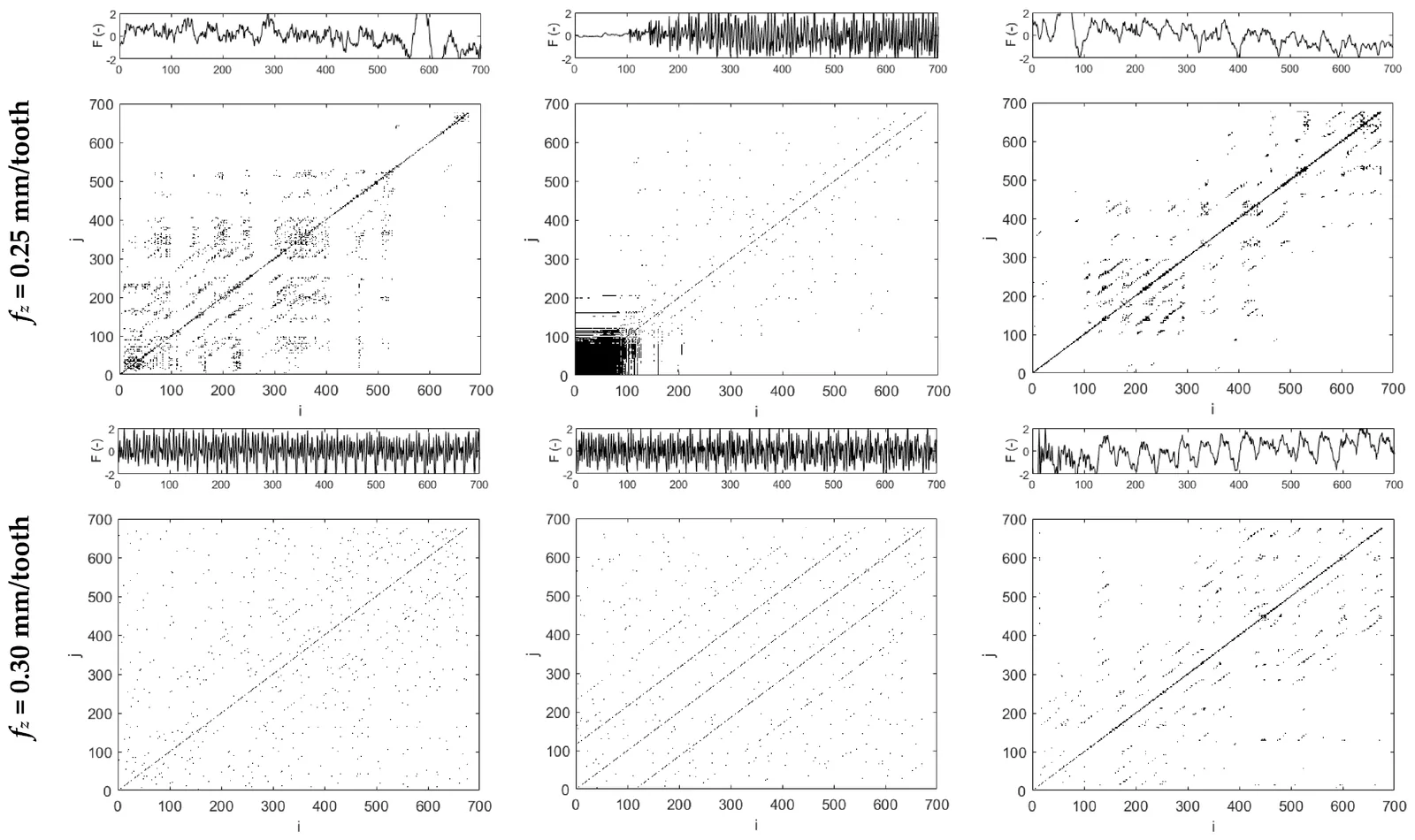
Recurrence plots for various tool holders at different feed per tooth.

The results of the recurrence analysis for variable feed rate values are presented in Figure 9. At feed rates up to *f_z_* = 0.15 mm/tooth, the recurrence plots indicate non-stationary vibration behavior. Increasing the feed per tooth clearly stabilizes the milling process; at *f_z_* = 0.2 mm/tooth, the vibrations distinctly take on a periodic character. Subsequently, at a feed of *f_z_* = 0.25 mm/tooth, additional fluctuations reappear particularly noticeable with the shrink-fit tool holder. Further increasing the feed per tooth once again stabilizes the milling process. Across all recurrence plots, the behavior is more favorable for the hydraulic tool holder, thereby confirming the conclusions drawn from the analyses in the preceding subsections.

## 4. Discussion

The analysis performed provided information on the dynamics of the trochoidal milling process of AZ31 alloy. The amplitude measures (P-P and RMS) reveal a strongly non-monotonic response to cutting speed. A local maximum in vibration amplitude occurs at *v_c_* = 600 m/min, followed by a decrease at *v_c_* = 800 m/min, whereas the highest amplitudes are recorded at *v_c_* = 1000 m/min. This behavior indicates a frequency-dependent dynamic response of the machining system. At *v_c_* = 600 m/min, the simultaneous minimum in CMSE suggests a more regular vibration signal dominated by periodic components. This combination is consistent with possible dynamic amplification, potentially including a resonant contribution. However, without an independent characterization of the system’s modal properties, resonance cannot be confirmed conclusively. CMSE nevertheless provides complementary information to conventional amplitude measures by revealing changes in signal irregularity that cannot be inferred from P-P or RMS values alone.

From a technological standpoint, comparison of the shrink-fit and hydraulic holders reveals a clear trade-off between vibration amplitude and signal complexity. The hydraulic holder generates lower vibration amplitudes than the shrink-fit holder, especially at higher cutting speeds and across the entire investigated feed range, while exhibiting slightly higher CMSE. This apparent discrepancy is not contradictory, as CMSE quantifies the temporal irregularity of a signal rather than its vibration amplitude or spectral bandwidth directly. In the hydraulic holder, small relative motions within the oil-filled clamping chamber may dissipate mechanical energy through viscous effects. Such damping can attenuate dominant resonant and harmonic components and, in terms of the frequency response, reduce and broaden resonant peaks. Consequently, once the more pronounced periodic components are suppressed, low-amplitude, non-periodic fluctuations may constitute a relatively larger proportion of the residual signal, increasing its temporal irregularity and, consequently, its CMSE despite lower P-P and RMS values. This interpretation is qualitatively consistent with the FFT results, which show fewer harmonics with lower amplitudes for the hydraulic holder, particularly in the mid-frequency range. However, the present results do not directly demonstrate that high-frequency vibration energy is redistributed over a broader spectrum, since CMSE is not a direct measure of spectral bandwidth and the frequency spectra were not quantitatively assessed in terms of bandwidth or spectral entropy. Therefore, the elevated CMSE observed for the hydraulic holder is interpreted as a possible consequence of the attenuation of dominant vibration components and the resulting increase in the relative irregularity of the measured signal, rather than as evidence of increased vibration severity or confirmed broadband energy redistribution. Previous modal and operational vibration analyses have likewise reported higher damping rates for hydraulic-expansion tool holders than for shrink-fit holders [35]. The shrink-fit holder, despite its higher static joint stiffness, generates the highest vibration amplitudes among the three solutions under most of the investigated conditions. This indicates that, in the present case, the dynamic response is influenced not only by joint stiffness but also by the damping characteristics of the tool–holder system. It is also worth noting that the scatter of the P-P results generally remains small (below 2.5%), but shows an increasing trend with both increasing cutting speed and increasing feed per tooth, particularly for the hydraulic holder as a function of feed. This suggests increasing variability in the dynamic response of the machining system as the machining parameters are intensified.

From a practical standpoint, *v_c_* = 400 m/min and *v_c_* = 800 m/min are comparatively favorable operating conditions within the investigated range, as they combine relatively low vibration amplitudes with distinct signal-complexity characteristics. The condition *v_c_* = 600 m/min warrants caution because the local amplitude maximum coincides with the lowest CMSE, indicating a relatively regular, periodic-dominated vibration response. The cutting speed *v_c_* = 1000 m/min should also be avoided because it produces the highest vibration amplitudes in the cutting speed series. In terms of feed per tooth, lower values (*f_z_* = 0.05–0.15 mm/tooth) are favorable for minimizing vibration amplitudes, despite the accompanying higher CMSE. Under these conditions, elevated entropy does not coincide with high vibration amplitude and should not be interpreted as an independent indication of an unfavorable dynamic state. By contrast, a feed of *f_z_* = 0.30 mm/tooth should be avoided because it generates the highest vibration amplitudes in the entire series, together with CMSE clearly above the minimum observed at *f_z_* = 0.20–0.25 mm/tooth.

This result also points to the practical value of jointly applying several signal-evaluation methods in process-monitoring systems. Exceeding a vibration-amplitude threshold alone does not make it possible to distinguish a state that is unfavorable for structural reasons (resonance, low entropy, energy concentrated in a narrow frequency band) from a state of comparable energy but of a more stochastic character (higher entropy), which may be associated with markedly different consequences for tool life and machined-surface quality. The simultaneous, joint observation of several types of indicators could, in the future, form the basis of a more sensitive system for detecting unfavorable dynamic process states than the use of a single threshold P-P or RMS value alone.

The use of FFT analysis and recurrence plots enables a deeper understanding of the vibration characteristics during the machining process. FFT analysis revealed that changes in cutting speed give rise to additional harmonic components, which can adversely affect the process. However, the smallest changes in the number of harmonics were observed with the hydraulic tool holder. Based on this, and consistent with earlier findings, it can be concluded that the use of this type of tool holder is recommended.

Recurrence analysis made it possible to identify the nature of the vibrations occurring during the machining process. The results for all tool holders reflect the harmonic nature of the process; however, this method revealed additional fluctuations when a shrink-fit holder was used. The observed phenomenon indicates a more complex vibration pattern, which under milling conditions can more easily transition into unsteady (less predictable) states.

When comparing the results presented in this paper, which utilize a trochoid trajectory, with those of an earlier publication on a straight-line trajectory [27], it can be observed that changes in *v_c_* and *f_z_* have a significant effect on vibrations. The greatest increase in vibration acceleration is observed as fz increases. This range spanned from 0.99 to 29.24 m/s^2^ for P-P, from 2.97 to 58.30 m/s^2^ for Peak, and from 0.99 to 29.24 m/s^2^ for RMS. Conversely, an increase in *v_c_* leads to an approximately linear increase in the values of the analyzed vibration parameters. The analyzed measures were as follows: P-P—6.89–32.69 m/s^2^; Peak—3.44–16.35 m/s^2^; RMS—1.43–8.39 m/s^2^. The type of tool holder also influences the magnitude of the generated machining vibrations, but to a much lesser extent than changes in the process parameters. CMSE analysis showed that the recorded time series are characterized by a relatively low level of signal irregularity. The vibration acceleration signals were characterized by lower uncertainty, and their entropy value decreased as the scale factor increased. CMSE generally does not exceed 2.2 for *v_c_* and 2.4 for *f_z_*, with higher CMSE values corresponding to lower scale factor values. The FFT analysis performed reveals high stability of the machining process. However, during milling at the highest *v_c_* and low *f_z_*, a susceptibility to chatter-type vibrations is observed.

## 5. Conclusions

- The amplitude of the vibration-acceleration signal (P-P, RMS) varies with cutting speed in a clearly non-monotonic manner, reaching its lowest values at *v_c_* = 400 m/min. FFT analysis revealed that at the lowest cutting speed, significantly fewer harmonic components are present with shrink-fit and hydraulic tool holders, particularly in the mid-frequency range. The local amplitude maximum and accompanying minimum of CMSE at *v_c_* = 600 m/min indicate a more regular, periodic-dominated vibration response and suggest possible frequency-dependent dynamic amplification. From the point of view of minimizing vibration, favorable cutting speeds are *v_c_* = 400 m/min and *v_c_* = 800 m/min, whereas *v_c_* = 600 m/min (elevated amplitude accompanied by the lowest CMSE) and *v_c_* = 1000 m/min (the highest amplitude in the entire series) should be avoided.
- An increase in feed per tooth leads to a systematic increase in vibration amplitude (by 16–45% for P-P and 40–76% for RMS), accompanied by a decreasing course of CMSE over practically the entire feed range studied. This opposite direction of change in the two indicators indicates that, as feed increases, the contribution of deterministic, periodic force excitation to the vibration signal increases, at the expense of a decreasing relative contribution of stochastic noise generated by the tool-clamping system. In terms of minimizing vibrations, the decisive criterion is vibration amplitude with the lowest values, *f_z_* = 0.05–0.15 mm/tooth, being the most favorable. The accompanying decrease in CMSE with increasing feed does not constitute an additional unfavorable indication here, owing to the increasing contribution of deterministic cutting force; the only exception is the ER holder.
- Comparison of the holders reveals a trade-off between vibration energy and signal complexity. The hydraulic holder generates a lower amplitude than the shrink-fit holder (by 3–15% in the cutting speed series and 14–25% in the feed series), but at the cost of a somewhat higher CMSE (by 4–21%). Owing to the greater practical significance of vibration amplitude, the hydraulic holder is identified as the preferred solution in terms of process dynamics. The ER holder, in turn, generates a signal with several-times-higher CMSE, owing to its more complex design.
- RP analysis showed that milling with the tested tool holders was characterized primarily by periodic vibrations, with frequencies shifting as process parameters increased. Certain deviations were observed when using the shrink-fit tool holder; in this case, additional fluctuations appeared, signaling a transition from periodic vibrations to other, less predictable types that can lead to detrimental self-excited vibrations.
- An examination of measurement signals using FFT and RP analysis demonstrated that the use of hydraulic tool holders is justified, especially at higher machining parameters. Due to their design, these tool holders provide significant vibration damping, a feature that favorably enhances milling stability.

## Figures and Tables

**Table 1:** ANOVA for Peak-to-Peak.

Effect	SS	DoF	MS	*F*	*p*	Effect	SS	DoF	MS	*F*	*p*
*v_c_*	596.01	3	198.67	30,576.70	0.00	*f_z_*	48.41	5	9.68	1197.11	0.00
Holder	8.95	2	4.47	688.39	0.00	Holder	27.18	2	13.59	1680.48	0.00
v_c_* Holder	9.28	6	1.55	238.13	0.00	f_z_* Holder	13.25	10	1.33	163.87	0.00

## Data Availability

The original contributions presented in this study are included in the article. Further inquiries can be directed to the corresponding author.
